# Successful Treatment of Chronic Mucocutaneous Candidiasis Caused by Azole-Resistant *Candida albicans* with Posaconazole

**DOI:** 10.1155/2011/283239

**Published:** 2010-12-01

**Authors:** Davide Firinu, Orietta Massidda, Maria Maddalena Lorrai, Loredana Serusi, Monica Peralta, Maria Pina Barca, Paolo Serra, Paolo Emilio Manconi

**Affiliations:** ^1^Department of Internal Medicine, Allergy and Clinical Immunology, Azienda Ospedaliero Universitaria, University of Cagliari, SS 554- Bivio Sestu, 09042 Monserrato, Cagliari, Italy; ^2^Department of Biomedical Sciences and Technologies, Medical Microbiology, University of Cagliari, 09100 Cagliari, Italy

## Abstract

Refractory or recurrent infections of skin, nails, and the mucous membranes are clinical signs of chronic mucocutaneous candidiasis, frequently associated with immunological defects. Here we describe a 39-years-old female patient, with familial CMC, that presented with an extensive infection caused by an azole-resistant *Candida albicans* isolate, successfully treated with posaconazole.

## 1. Introduction

Chronic mucocutaneous candidiasis (CMC) is a persistent or refractory/recurrent infection of the skin, nails, and mucous membranes, most commonly caused by *Candida albicans*, that can be related to a variety of disparate clinical conditions, yet to be fully identified [1]. Although different underlying diseases predispose to CMC, they are frequently associated with primary or secondary immunodeficiencies. Regarding secondary causes, HIV infection is common, although other etiologies are known [1]. Among inherited causes, sporadic, autosomal dominant (MIM 114580), and autosomal recessive (MIM 212050) forms of CMC have been described. Moreover, similar clinical patterns of candidiasis are shared by other primary immunodeficiencies, mainly APECED (MIM 240300) [2] and autosomal-dominant hyper-IgE syndrome (MIM 147060) [3].

The availability of azoles (e.g., clotrimazole, ketoconazole, itraconazole, and fluconazole) represented a dramatic improvement in the treatment of all forms of CMCs. However, following the use of these drugs, *C. albicans* strains resistant to azole antifungals have been subsequently isolated [4], requiring novel therapeutic options. These include flucytosine, amphotericin B, the newest azoles and, more recently, echinocandins.

Here we describe a case of a familial CMC, characterized by a refractory infection caused by *C. albicans* resistant to azoles, including voriconazole, successfully treated with posaconazole that to our knowledge has not yet been reported to treat these forms of candidiasis.

## 2. Case Presentation

A 39-year-old female patient was referred to our Center in 2009, presenting a history of recurrent infections with involvement of mucosa, nails, and skin caused by *C. albicans*. At the onset, when the patient was 2 years old, the fungal infection started on the face and nails and progressively diffused to other cutaneous and mucosal tissues. At 3 years of age, oral thrush, labial fissures, and cutaneous erythematous desquamating patches developed and have persisted since then. Clinical samples constantly revealed the presence of *C. albicans*. The clinical diagnosis of familial CMC was posed. The patient received courses of systemic treatment with clotrimazole. However, recurrence of candidiasis occurred shortly after halting antifungal therapy. The patient's family included unaffected parents as well as two unaffected brothers and two sisters, while another brother, affected by a severe form of CMC, died when he was 6 years old of fulminant hepatitis. 

**Figure 1:**
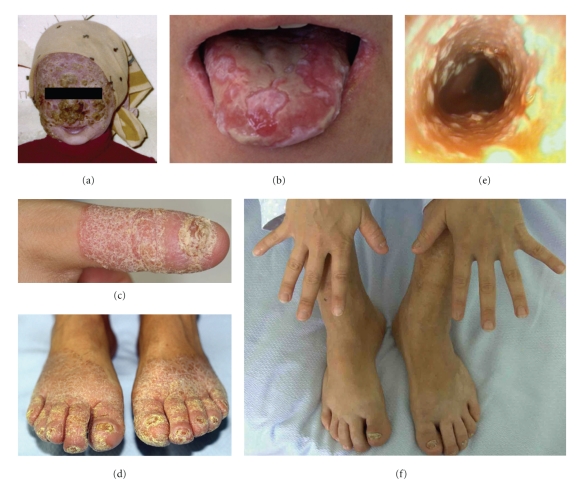
(a) Extensive dermatophytosis, caused by *M. canis* at 6 years of age. (b–e) Clinical presentation of *C. albicans* infection at the time of admission: (b) whitish and yellowish plaques on the tongue and perleche, secondary to candidal infection; (c) skin and nail of the right thumb, (d) skin and nails of the feet; (e) endoscopy showing severe esophagitis. (f) Hands and feet after 8 months of treatment with oral posaconazole, showing a complete regression of skin and nails candidiasis.

At 6 years of age, the patient experienced a massive erythematous-desquamating dermatosis involving the face, limbs, nails, and the oral, conjunctival and genital mucosa. In addition, she developed a disfiguring dermatophytosis, caused by *Microsporum canis*, detected in the squamous samples, restricted to face and scalp (Figure 1(a)). Treatment with clotrimazole and griseofulvine led to a slow, albeit complete, recovery, although she developed alopecia of eyelashs, eyebrows, and scalp. Throughout her life she experienced several recurrent infections by *C. albicans*, for which she received long-term courses of different antifungals, such as clotrimazole, miconazole, and ketoconazole and as soon as they became available, fluconazole and itraconazole. The therapies with azoles were overall successful to control recurrent candidiasis. Nevertheless, since 2005 a progressive decrease in the susceptibility of *C. albicans* isolates to azoles, parallel to a worsening of her symptoms, required an increased dosage of these drugs.

When the patient was admitted to our hospital in June 2009, she presented with an extensive candidiasis of the mouth, hands, and feet (Figures 1(b), 1(c), and 1(d)). In addition, she complained of dysphagia and a weight loss of 10 kg in 2 months. Specimens from cutaneous, pharyngeal, and buccal swabs were positive for *C. albicans, *while a nasal swab was negative. In addition to *C. albicans*, cultures of all specimens grew also *Escherichia coli *and* Enterobacter cloacae.* All *C. albicans* isolates showed the same susceptibility profiles to antifungal drugs, as detected with antimycograms. In particular, they were resistant to nystatin, fluconazole, itraconazole, voriconazole but sensitive to posaconazole, flucytosine, amphotericin B and to echinocandins. Esophagogastroduodenoscopy (EGD) showed an esophageal and duodenal candidiasis (Figure 1(e)). Cancer was excluded by biopsy. Screening investigations including full blood count, routine blood chemistry, C-reactive protein, liver function tests, protein electrophoresis, levels of ferritin, urea and electrolytes, IgA, IgG, IgE, IgM, C3, C4. and tests for thyroid, parathyroid, adrenal, and HIV antibodies were performed. Lymphopenia was present (800–1,200 cells/mm^3^) with lymphocyte count upon admission of CD45+ 888 cells/mm^3^ (1,600–2,400), CD3+ 772 cells/mm^3^ (960–2500), CD4+ 484 cells/mm^3^ (540–1,400), CD8+ 238 cells/mm^3^ (270–930), CD19+ 40 cells/mm^3^ (90–400), and CD16+56+ 52 cells/mm^3^(90–590). Erythrocyte sedimentation rate was 65 mm/hour and all the other laboratory tests were normal, consistent with the clinical presentation of the case. Antinuclear Antibodies (ANAs) were positive, but endocrinopathies or other defined autoimmune diseases were excluded. In particular, we did not find an association between CMC and hypothyroidism. The thyroid function was normal, as determined by the normal values of FT3, FT4 and TSH (4.6 pg/mL; 16.3 pg/mL and 2.55 *μ*IU/mL, resp.) and negativity of antithyroid antibodies. Moreover, thyroid physical examination and ultrasonography were normal. The autoimmune polyendocrinopathy-candidiasis-ectodermal dystrophy (APECED) [2], common in the Sardinian population [5], was ruled out by genetic analysis of the AIRE gene, that showed a wild-type sequence. Hyper-IgE syndrome was excluded by clinical features [3] and IgE levels were normal (4.63 kU/L). HIV infection was ruled out by negative serological and viral RNA-based tests. Diabetes mellitus, neoplasias, immunosuppressive treatments, and other clinical conditions commonly associated with *Candida *infection were also ruled out.

The diagnosis of familial CMC was confirmed and a treatment with amphotericin-B (50 mg/day IV) for 2 weeks was promptly started for the treatment of severe resistant skin, mouth, and esophageal candidiasis, resulting in an improvement of dysphagia. Orally administered posaconazole (400 mg twice per day) replaced amphotericin-B, resulting in a significant regression of the dysphagia as well as a clear improvement of the cutaneous candidiasis after 2 weeks of treatment. Onychomycosis regressed partially and more slowly during the following months (Figure 1(f)). After 2 months, the dosage of oral posaconazole was reduced to 200 mg once a day, without a relapse of clinical symptoms. 

The therapy was temporarily discontinued after 3 months. About 2 weeks later, a relapse of candidiasis was observed, although it was limited to the oral mucosa, with no involvement of other sites. Posaconazole was then started again at a dosage of 200 mg, three times per day for a month with a 15-day discontinuation. As maintenance therapy, this regimen is being repeated cyclically with no observable side effects.

## 3. Discussion

Although CMCs can arise from a variety of clinical conditions, they may disclose rare primary immunodeficiencies, reflecting defects in the first line of host defence against fungi. 

Interestingly, Glocker et al. [6] and Fewerda et al. [7] recently reported the first monogenetic defects in humans, who presented with the clinical features of CMC and other mycoses, caused by mutations in the genes encoding CARD9 (MIM 607212) and Dectin-1 (MIM 613108). These defects are linked to the role that these proteins play in the activation of the multifaceted Th17 lymphocytes and their production of interleukins (e.g., IL-17 and IL-22) for epithelial host defense against fungal infection. Animal models suggested that, a multipart pathway, starting from the yeast transmembrane pattern recognition receptor Dectin-1 on epithelial cells and phagocytes, leads to the activation of the CARD9 signaling complex that produces cytokines that initiate differentiation of CD4+ T lymphocytes toward the Th17 phenotype, crucial for the adaptive antifungal immunity [8]. In addition, the discovery in humans of autoantibodies against IL-17 and IL-22, as recently observed in APECED [9, 10] further supports the role of Th17 in host defense against *Candida*. Finally, new evidence supporting the role of IL-22 in protection from candidiasis has been reported [11, 12]. Therefore, impairment of the Th17 lymphocytes and/or their cytokines appears to be the common denominator of genetically heterogeneous but clinically related disorders.

CMCs are characterized by persistent refractory/recurrent infections, commonly caused by *C. albicans*, and occasionally complicated by dermatophytosis [1], and therefore require long-term and extensive antifungal treatment. Until the late 1980s, patients with chronic candidiasis were typically treated only when symptomatic. From the late 1980s, the availability of new azoles (e.g., clotrimazole, ketoconazole, itraconazole and fluconazole) represented a dramatic improvement in the management, prophylaxis, and treatment of these forms of CMC. However, following the use of these drugs, a decrease in the susceptibility of the *C. albicans* strains to azole antifungals became common and, in the treated patients, the therapeutic response started failing with the consequent development of refractory candidal infections [4]. Other therapeutic options include flucytosine, amphotericin B, and the newest azoles, such as voriconazole and posaconazole, and more recently echinocandins. Different studies have reported successful treatments of CMC caused by azole-resistant isolates of *Candida spp*., with systemic amphotericin B [13–15], voriconazole [16], or echinocandins [17, 18].

In the case of our patient, besides nails, skin, and oral mucosa, an extensive esophageal and duodenal azole-resistant *C. albicans* infection was also present. While topical therapy is ineffective, oral or intravenous azoles represents first-line treatments of these forms [19]. Nevertheless, *C. albicans *resistant to azoles, including voriconazole, leaves clinicians with few therapeutic options, further limited by toxicity and route of administration. Although amphotericin B or echinocandins are primarily recommended in the treatment of esophageal infection caused by azole-refractory *Candida spp*. [19], difficulties regarding the intravenous route of administration and/or toxicity, particularly for prolonged use, may be problematic. Among the possible choices, posaconazole has the advantage of being both safe in long-term use and orally administrable [19, 20]. For this reason, we chose to treat our patient with oral posaconazole, even though amphotericin B was promptly administered, given the patient's conditions and the time required to obtain posaconazole. The treatment was very well tolerated and brought complete control of the *C. albicans* in all sites of infection. An intermittent posaconazole administration scheme is currently undergoing, aiming to reduce the relapse rate and, possibly, the risk of resistance.

## 4. Conclusions

Although in the case of our patient a specific genetic explanation for her hereditary primary immunodeficiency cannot yet be offered, posaconazole treatment of the chronic infection sustained by azole-resistant *C. albicans* was confirmed to be appropriate, given both the patient's clinical and microbiological history.
